# Right-Sided Congenital Diaphragmatic Hernia Caused by Hepatopulmonary Fusion

**DOI:** 10.1155/2020/8851341

**Published:** 2020-10-29

**Authors:** Sonal Patel, Jennifer Rael

**Affiliations:** Department of Pediatrics, Division of Neonatology, University of New Mexico, Albuquerque, NM, USA

## Abstract

**Introduction:**

Hepatopulmonary fusion is a very rare finding associated with right-sided congenital diaphragmatic hernia. With less than 50 reported cases, management and outcomes of hepatopulmonary fusion are poorly understood. This report highlights that clinical presentation is not a reliable indicator of outcomes in this rare disease. *Case Presentation*. A term neonate admitted for tachypnea and complete opacification of the right hemithorax was diagnosed with right-sided congenital diaphragmatic hernia. Preoperative respiratory support was minimal, and the only symptom exhibited was tachypnea. During surgical repair, fusion of the lung and liver were noted, consistent with a diagnosis of hepatopulmonary fusion. Postoperatively, the patient's pulmonary hypertension worsened and required extracorporeal membrane oxygenation.

**Conclusions:**

Many patients with hepatopulmonary fusion and only mild symptoms die postoperatively from severe pulmonary hypertension and progressive respiratory failure. Preoperative clinical status is not indicative of postoperative outcomes, and literature suggests that patients who require less support preoperatively have high mortality rates. The availability of ECMO for postoperative complications may be necessary in patients requiring repair of hepatopulmonary fusion.

## 1. Introduction

Congenital diaphragmatic hernia (CDH) occurs when the diaphragm fails to completely develop, resulting in herniation of abdominal contents into the chest cavity. The most common defect in the posterior lateral portion of the diaphragm results in a Bochdalek hernia. Morgagni hernias occur when the defect is in the central anterior portion of the diaphragm; these are often asymptomatic and will not be discussed here. Bochdalek CDH can result in a wide range of disease, from mild respiratory symptoms to severe pulmonary hypoplasia. It is estimated to occur in 1 in 3591 live births [1]. Left-sided CDH is more common than right-sided CDH, occurring in 75–90% vs. 10–15% of all CDH cases, respectively [2]. Hepatopulmonary fusion (HPF) is a unique and rare congenital malformation associated only with right-sided CDH. There are less than 50 reported cases, and HPF has an estimated incidence of 3 out of 1000 cases of CDH with a mortality rate of approximately 50% [3]. We present another case of HPF and discuss important patterns in the literature.

## 2. Case Presentation

**Figure 1:**
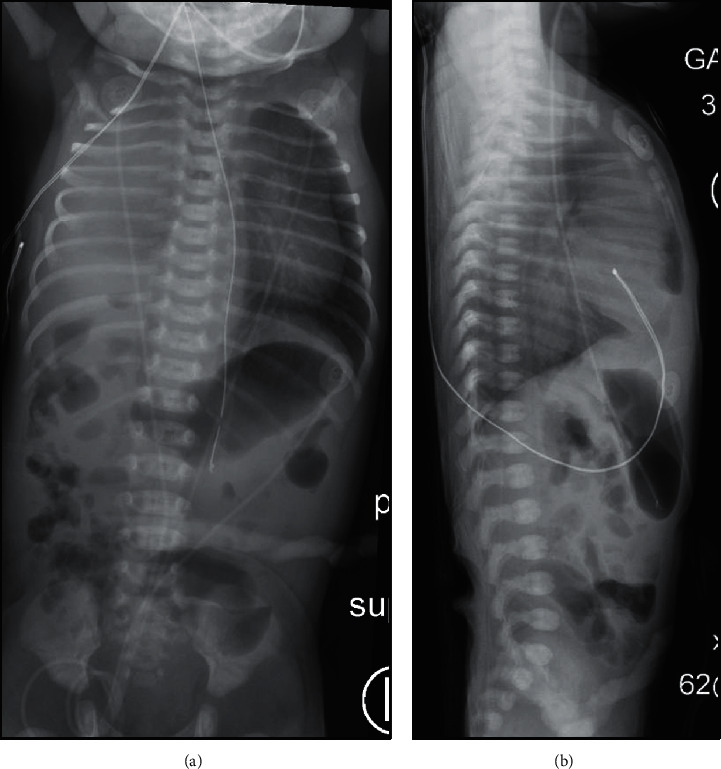
Radiographs upon admission. (a) Anterior-posterior view depicting complete opacification of right hemithorax with leftward mediastinal shift. (b) Cross-table lateral view depicting suspected hernia of abdominal contents into chest cavity.

A female infant was born at 37 weeks and 3 days in a community hospital via caesarean section for breech presentation to a 36-year-old gravida 2 para 1, now para 2, mother. Her mother received an ultrasound for dating that showed normal anatomy. Otherwise, her pregnancy was complicated by polyhydramnios and urinary tract infections. At birth, the patient required full resuscitation including positive pressure ventilation for 15 minutes and chest compressions for an unknown amount of time. APGAR scores were 1, 4, 7, and 9 at 1, 5, 10, and 15 minutes, respectively. She was placed on continuous positive airway pressure (CPAP) after resuscitation, and chest radiograph showed complete opacification of the right hemithorax. She was transferred soon after birth to a tertiary care center for respiratory distress. At 6 hours of life, she arrived to the tertiary care center's neonatal intensive care unit (NICU) with tachypnea on CPAP 6 and 40% oxygen requirement. Initial exam revealed absent breath sounds on the right, decreased breath sounds on the left with good air entry, and no retractions. Radiographs upon admission are shown in Figure 1. An echocardiogram performed soon after admission showed severe persistent pulmonary hypertension (PPHN) with moderately dilated right atrium and ventricle, large patent ductus arteriosus, hypoplastic right pulmonary artery, and right chest mass. Due to concern for pulmonary effusion, a thoracentesis was performed without return of fluid.

**Figure 2:**
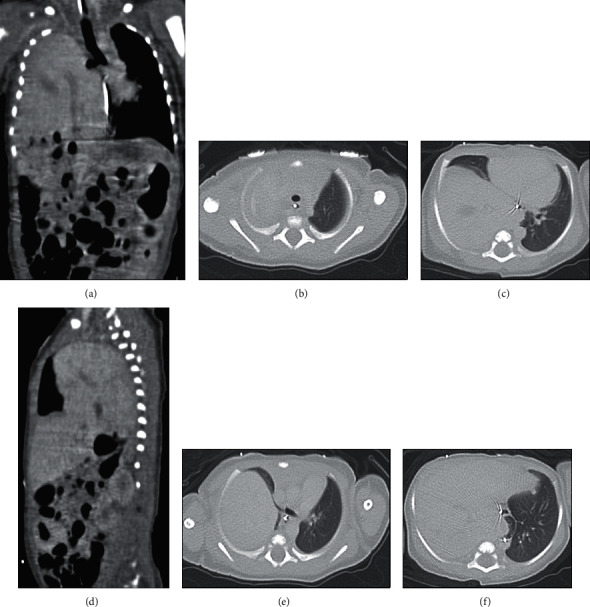
Computed tomography scans one day after birth. (a) Coronal view of the liver in the right hemithorax extending to the apex with leftward mediastinal shift. (b) Axial images depicting the apex of the lungs, with the right lung apex completely compressed by the liver. (c) Axial view showing the leftward mediastinal shift and the extent of aeration of the right middle lobe. (d) Sagittal view of right lung at the level of greatest aeration. (e) Axial view depicting the right mainstem bronchus and cardiothymic shift. (f) Axial view suggesting possible aplasia of the right lower lobe.

An ultrasound performed on day of life (DOL) 1 showed liver in the right hemithorax with leftward cardiothymic shift, concerning for CDH. Computed tomography (CT) confirmed large right-sided diaphragmatic hernia containing the entirety of the liver and gallbladder, as well as the hepatic flexure of the colon. The liver dome extended to the apex of the right hemithorax (Figures 2(a) and 2(b)). The upper lobe of the right lung was significantly compressed, and there was only partial aeration of the right middle lobe (Figures 2(c) and 2(d)) with leftward cardiothymic shift (Figure 2(e)). The right lower lobe bronchi were not seen, concerning for aplasia of that lobe (Figure 2(f)).

The patient was weaned from CPAP on DOL 2 and placed on high-flow nasal cannula at 5 liters with 21% oxygen. Her tachypnea remained unchanged from birth. She remained on 5 liters of high flow until surgery secondary to her tachypnea, but was otherwise well-appearing. She remained NPO from birth. The infant was taken for corrective surgery on DOL 7. A right subcostal incision was carried down through subcutaneous tissues and anterior rectus sheath using electrocautery. The underlying rectus muscle divided with cautery, and the posterior sheath sharply opened into the abdomen. After the small bowel and colon were eviscerated, the liver was seen extending through the large diaphragmatic defect. There did appear to be good diaphragm in the lateral aspect. As the liver was being reduced, significant adhesions between the lung and what appeared to be a sac in the hernia were noted. The sac was mobilized from the edge of the diaphragm circumferentially, and fusion of the lung was identified. The lung and liver were densely adherent without an easily identifiable separation plane. The adhesions of the liver and lung to the sac were then mobilized and dissected starting medially, with clear identification of the hepatic veins, and the suprahepatic vena cava, the lung, and liver were able to be completely separated from each other. At this point, the liver was reduced into the abdomen, and on inspection, the lung was noted to be quite hypoplastic. The edges of the diaphragm were free, but the sac seemed adherent into the chest. Medially, this tissue was overlying the area of the suprahepatic cava and was not removed secondary to its proximity to this vessel. The repair of the diaphragm consisted in primary repair laterally. The medial third of the defect required the use of a GORE-TEX patch for closure. A surgical chest tube was left in place, and the patient was returned to the NICU. Of note, immediately postoperatively, the patient was unstable with the chest tube to suction, thought secondary to mediastinal shift, and the chest tube was therefore placed to gravity.

After returning to the NICU, the patient became increasingly difficult to ventilate. On evaluation, a large blood clot was eventually found in the endotracheal tube during tube exchange, after which ventilation significantly improved. The patient was placed on high-frequency oscillator ventilation immediately postoperatively and weaned to conventional ventilator the same day. She was started on 20 parts per million (ppm) of inhaled nitric oxide (iNO) during surgery, and she was weaned to 10 ppm a few hours after surgery. Roughly two hours after surgical repair, her mean arterial pressures decreased to 20 mmHg. Dopamine was subsequently started, with epinephrine added later. She remained on 40% oxygen throughout the night.

On postoperative day (POD) 1, the patient's oxygen requirement increased to 65% in the morning and remained there the majority of the day. An echocardiogram revealed suprasystemic PPHN, increased from half-systemic preoperatively. The iNO was increased back to 20 ppm, and milrinone was started. The infant progressively worsened and required high-frequency jet ventilator as her oxygen requirement continued to increase. Over the course of that day (POD 1), the infant's oxygen index increased from 26 to 32 with saturations in the 80s on 100% oxygen. The decision was made to cannulate for extracorporeal membrane oxygenation (ECMO). Because the venovenous cannula was thought to be too big for this patient's size, she was placed on venoarterial ECMO. During ECMO, milrinone, dopamine, and epinephrine were able to be weaned off. Prior to, during, and after ECMO, the patient was showing signs of disseminated intravascular coagulation and required multiple transfusions of packed red blood cells, fresh frozen plasma, and platelets. ECMO was discontinued after approximately 72 hours on POD 4. Surgical chest tube was removed on POD 5. She was extubated on POD 6 (DOL 13). Milrinone was restarted on DOL 12 after the first echocardiogram off of ECMO suggested half-systemic PPHN. The milrinone was stopped on DOL 14, and iNO was weaned the following day and completely off by DOL 18. Sildenafil was started on DOL 16 for long-term treatment of PPHN. The patient was started on trophic feeds on DOL 15 and reached full feeds 7 days later. The patient was slowly weaned off respiratory support, eventually to low-flow nasal cannula by DOL 33. Due to tachypnea with average respiratory rate approximately 70–80 breaths per minute, the patient had difficulty feeding by mouth. The G-tube was placed on DOL 56. The patient was discharged home on DOL 64 with sildenafil and minimal oxygen support.

**Figure 3:**
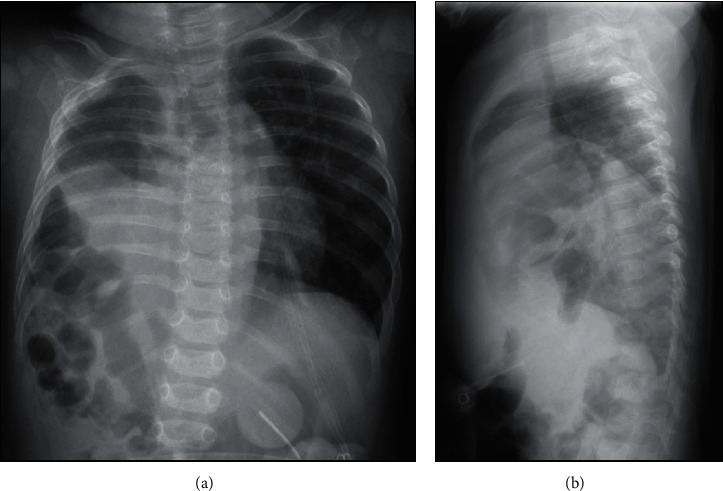
Follow-up radiograph at 5 months of age. (a) Anterior-posterior view suggesting minimal right lung aeration. (b) Lateral view showing the different locations of the two diaphragms, suggesting profound right pulmonary hypoplasia.

Follow-up radiographs at 5 months of age continue to indicate right pulmonary hypoplasia with high diaphragm (Figure 3).

## 3. Discussion

Clinically, this patient appeared to have a mild form of right-sided CDH with tachypnea as the only obvious symptom. The half-systemic PPHN was asymptomatic, as her oxygen requirement was 21%, and her preductal and postductal oxygen saturations were not different. Intraoperatively, it was discovered that this patient had HPF. Postoperatively, the patient's pulmonary hypertension worsened to the point that ECMO was required approximately 30 hours after repair. This degree of postoperative illness severity was not expected based on preoperative symptoms. However, thorough literature review indicates that this is not an unusual course for many patients with HPF. To our knowledge, there are 37 reported cases in 21 published reports of HPF in the English language; this excludes two cases published in 1977 and called “pseudosequestration” [4]. Of those, 9 cases are similar to the one reported here [5–13], 10 cases are not similar in patient presentation, comorbidities, and/or management [14–21], 17 cases do not include enough information for comparison [3, 22, 23], and 1 case is a secondary HPF after initial repair of right-sided CDH [24]. Of these 37 cases, there were 19 deaths and 18 survivors.

The nine cases similar to the one reported here all include patients who presented with some degree of respiratory distress within hours of birth and were repaired in a similar way, without significant other comorbidities. Of these nine cases, two patients were intubated at birth and required significant support for the management of ventilation and PPHN. Of the remaining seven patients, one patient was intubated at one day of age for respiratory distress and treated for PPHN; two patients were intubated secondary to the diagnosis of CDH without reported treatment for PPHN; and five patients did not require preoperative intubation and did not show clinical signs of PPHN. All seven of these patients with milder preoperative disease died postoperatively within hours to days, while the two patients requiring more support from birth survived (Table 1). None were placed on ECMO.

The mortality rate of HPF remains very high, even in the setting of mild preoperative clinical findings. The differential diagnosis of right-sided CDH should always include HPF, a diagnosis of which would significantly alter the surgical intervention, management, and outcomes. Preoperative diagnosis of HPF, although difficult, is possible, and efforts should be made in all cases of right-sided CDH to rule out HPF before surgery. Corrective surgery for patients with HPF, regardless of clinical presentation, should be performed at a center prepared to do ECMO postoperatively.

## Figures and Tables

**Table 1:** Summary of nine similar cases.

First author	Age at presentation	Intubated before surgery	Pulmonary hypertension	Age at repair	Survived	Age at death
Katz et al. [5]	10 hours	Yes^*∗*^	Not reported	6 days	No	10 days

Keller et al. [6]	Birth	Yes	Suprasystemic	8 days	Yes	—

Saurabh et al. [7]	13 hours	No	Not reported	Not reported	No	11 days

Hamilton et al. [8]	3 months^§^	No	Not reported	3 months	No	3 months

Olenik et al. [9]	Birth	Yes	Yes	2 days	Yes	—

Laamiri et al. [10]	21 hours	Yes^*∗*^	Not reported	Not reported	No	Not reported

Jain et al. [11]	2 months${}^{\begin{array}{c} †\\ † \end{array}}$	No	No	2 months	No	12 hours after repair

Almaramhy [12]	Birth	No	No	2 days	No	5 days

Kerkeni et al. [13]	1 day	Yes	Yes	3 days	No	7 days

^*∗*^Intubated only after diagnosis of CDH, not for respiratory distress. ^§^Tachypnea at birth; presented with dyspnea, cough, and wheezing. ${}^{\begin{array}{c} ‡ \end{array}}$Tachypnea presented early in life; third evaluation for tachypnea.

## Data Availability

No data were used to support this case report.

## Abbreviations

CDH: Congenital diaphragmatic hernia
HPF: Hepatopulmonary fusion
CPAP: Continuous positive airway pressure
NICU: Neonatal intensive care unit
PPHN: Persistent pulmonary hypertension
DOL: Day of life
CT: Computed tomography
ppm: Parts per million
iNO: Inhaled nitric oxide
POD: Postoperative day
ECMO: Extracorporeal membrane oxygenation.
